# Uremic Pruritus Is Not Associated with Endocannabinoid Receptor 1 Gene Polymorphisms

**DOI:** 10.1155/2016/3567527

**Published:** 2016-02-29

**Authors:** Monika Heisig, Łukasz Łaczmański, Adam Reich, Felicja Lwow, Jacek C. Szepietowski

**Affiliations:** ^1^Department of Dermatology, Venereology and Allergology, Wroclaw Medical University, 1 Chalubinskiego Street, 50-368 Wroclaw, Poland; ^2^Department of Endocrinology and Diabetology, Wroclaw Medical University, 4 Pasteura Street, 50-367 Wroclaw, Poland; ^3^Department of Health Promotion, University School of Physical Education, 35 Paderewskiego Street, 51-612 Wroclaw, Poland

## Abstract

Uremic pruritus (UP) is a frequent and bothersome symptom in hemodialysis patients. Its etiology is not fully understood and that is why there is no specific treatment. The endocannabinoid system plays a role in many pathological conditions. There is reliable evidence on the association between cannabinoid system and pruritus. In our study, we aimed to evaluate whether genetic variations in the endocannabinoid receptor 1 (CNR1) gene can affect UP. The rs12720071, rs806368, rs1049353, rs806381, rs10485170, rs6454674, and rs2023239 polymorphisms of the CNR1 gene were genotyped in 159 hemodialysis patients and 150 healthy controls using two multiplex polymerase chain reactions and the minisequencing technique. No statistically significant relationship was found in any of the evaluated genotypes between patients with and without UP, even after excluding patients with diabetes and dyslipidemia. There were no differences between patients with UP and the control group. However, in the group of all HD patients, a significantly higher incidence of GA genotype and lower incidence in GG genotype in the polymorphism rs806381s were revealed versus the control group (*p* = 0.04). It seems that polymorphisms of the CNR1 gene are not associated with uremic pruritus.

## 1. Introduction

Uremic pruritus (UP) is a frequent phenomenon and it is regarded as one of the most bothersome symptoms in patients with chronic renal disease [1]. The prevalence of UP is still high and reported in around 40% to 50% [2, 3]. UP has an important impact on patients' quality of life and sleep, depression, and increased mortality [3, 4]. The pathogenesis of UP remains blurry, although many different factors have been indicated in the etiology of this symptom, including increased systemic inflammation, abnormal serum parathyroid hormone, calcium, and phosphorus levels, an imbalance in opiate receptors, a neuropathic process, or even skin dryness [4, 5]. This is why until now there is no specific treatment for patients with UP and many of the available therapeutic modalities are not satisfactory.

The endocannabinoid system (EC) has an effect on various physiological conditions and since its discovery at the end of twentieth century it has attracted much attention of researchers in different fields. It has already been proved that the EC system plays a role in insulin resistance, fat distribution, and metabolic disorders [6, 7]. Moreover, recent studies provided data on the significant role of the EC in the cutaneous physiology and pathology [8]. The EC has two identified receptors: CNR1 (endocannabinoid receptor 1) and CNR2 (endocannabinoid receptor 2). Both receptors have their ligands which interact with endogenous and exogenous cannabinoids [9]. The most well-known natural ligands of CNR1 are fatty acid amides (FAAs) or esters represented by anandamide (AEA), N-palmitoylethanolamine (PEA), N-oleoylethanolamine (OEA), or 2-arachidonoylglycerol (2-AG) [7, 10]. The first receptor is mainly expressed in central nervous system but also its presence has been detected in peripheral organs, including heart, lungs, gastrointestinal tract, liver, adrenal glands, bladder, and skin [8, 11], whereas CB2 receptor has been described predominantly in tissues and cells associated with immune system [8, 12]. The CNR1 gene can be changeably spliced resulting in modification of its second exon. As a result, there are polymorph forms of CNR1 gene [13] which may lead to dysregulation in some processes. There are data providing association between some of the CNR1 polymorphisms and abdominal obesity, metabolic syndrome, microvascular damage, polycystic ovary syndrome (PCOS), and nonalcoholic fatty liver disease [14, 15]. Some of the polymorphisms identified in the CB2 receptor gene have been associated with a risk of autoimmune disorders [16].

Our group has already confirmed the antipruritic effect of cannabinoids in the pilot study on patients with UP in which the application of a cream containing cannabinoid agonists (AEA and PEA) resulted in a significant reduction or even elimination of this symptom in most of the patients [17, 18]. We decided to evaluate further the impact of the EC on UP by analyzing the relationship between polymorphic variants of CNR1 gene and this symptom. To the best of our knowledge, the role of CNR1 gene polymorphism has not been evaluated yet, not only in UP, but also in other forms of pruritus.

## 2. Materials and Methods

### 2.1. Patients

The study was conducted in 159 patients on maintenance hemodialysis (HD) (62% males and 38% females). They were between 27 and 91 years of age (mean 63.0 ± 13.3 years). Patients were divided into “itch” and “no itch” groups. Patients were considered to have uremic pruritus if the itch appeared shortly before the onset of dialysis, or at any time afterwards without evidence of any other disease or drug intake that could cause pruritus. Patients were excluded if they had a prolonged pruritus caused by an additional disease. The control group consisted of 150 healthy people who were between 50 and 62 years of age (mean 55.5 ± 2.6 years). Participants were recruited from cohorts of patients admitted to three different hemodialysis centers in south-west Poland between September 2013 and February 2015. An informed consent to participate in the study was obtained from all patients. The study was approved by the Ethic Committee of Wroclaw Medical University (number 466/2013).

### 2.2. Genetic Studies

Whole genomic DNA was isolated from blood leukocytes using standard methods. CNR1 genotyping (rs12720071, rs806368, rs1049353, rs806381, rs10485170, rs6454674, and rs2023239) was performed by two multiplex polymerase chain reactions (PCR) and minisequencing described in details elsewhere [19, 20]. The specific primers used in the study are given in Table 1. Products were analyzed by an ABI 310 sequencer (Applied Biosystems). The size of the products was calculated using GeneScan 3.1 (Applied Biosystems).

### 2.3. Statistical Analysis

All data were analysed using Statistica 12.0 (Statsoft, Cracow, Poland). Means, standard deviations (SD), and minimum and maximum values as well as frequencies were calculated. Differences between analyzed groups were verified with *χ* ^2^ test. *p* values <0.05 were considered to be significant.

## 3. Results

The frequency of the genotypes of the CNR1 gene polymorphisms evaluated in patients with UP and without UP is shown in Table 2. No statistically significant difference was found in any of the evaluated genotypes between studied groups. Moreover, we still did not see any significant association between patients with and without UP after excluding individuals with diabetes and dyslipidemia (data not shown). Additionally, there were no differences between patients with UP and the control group. However, in the group of all HD patients, a higher incidence of GA genotype and lower incidence of GG genotype in the polymorphism rs806381s were revealed versus the control group (*p* = 0.04) (Table 3).

## 4. Discussion 

The role of the EC has attracted our particular attention for its influence on different skin conditions, including pruritus. There are data on significant relief of pruritus in different systemic diseases after the use of cannabinoid receptor agonists. Neff et al. [21] reported a relief in an unmanageable pruritus in a group of patients with cholestasis after the use of dronabinol. Furthermore, our group confirmed the antipruritic effect of cannabinoids in a pilot study on patients with UP in which the application of a cream containing cannabinoid agonists (AEA and PEA) resulted in a significant reduction or even elimination of this symptom in most of the patients [17, 18]. Another research with topical cannabinoid agonists confirmed their antipruritic properties in patients with prurigo, lichen simplex, or refractory pruritus [22]. In addition, it was found that suppression of the neuronal FAAH reduces the scratching response through the inhibition of AEA degradation and activation of CNR1 [8]. Moreover, there is a reliable evidence that endocannabinoids play also a role in modulation of pain perception and nowadays they are being considered as potential analgesic drugs [23, 24]. We already know that some pathways of itch and pain conduction can overlap [25] and this gave us another rationale to evaluate further the relationship between cannabinoid receptors and UP. The distribution and expression of CNR1 gene were confirmed by the study of Ständer et al. [26] found in skin biopsies taken from different body sites. In the skin, CB1 receptor was mainly expressed on cutaneous nerves in dermis and epidermis especially on skin nerve fibers and mast cells which again provides implications for an antipruritic but also anti-inflammatory and antinociceptive action of cannabinoid receptor agonists.

Our study suggests no associations between the CNR1 gene polymorphism and the presence of UP which, in the context of the aforementioned therapy strategies, seems to be rather unexpected. However, it has to be highlighted that cannabinoid receptor agonists are not fully specific ligands and they may regulate other mechanisms that play a role in the pathogenesis of pruritus. Although CNR1 seems to have predominant role in the cutaneous physiopathology, CNR2 has also been found in the skin, especially on large myelinated nerve fiber bundles of the superficial and deep reticular dermis, small unmyelinated nerves of the papillary dermis, and occasionally on nerves in epidermis [8]. Moreover, N-palmitoylethanolamine can downmodulate mast cell degranulation by interacting with CNR2 receptors which are additionally expressed on mast cells [27]. It has been shown that topical administration of cannabinoid receptor agonists, probably via CNR2 activation, significantly reduces histamine-induced itch and vasodilatation [28]. It has also been reported that cannabinoids can activate non-CNR1/CNR2 mechanisms. It was observed that cannabinoids may activate numerous other receptors, including PPARs (peroxisome-proliferators-activated receptors), which have already been reported to have an important role in pruritus pathogenesis, as their activation can significantly diminish itch in some skin diseases [29].

We are aware of the fact that many different factors can interfere with the polymorphism of CNR1 gene. That is why we also did our statistical analysis after excluding from the HD patients' group individuals with diabetes and dyslipidemia. However, it did not make any change in the final result. As EC is still not fully discovered, we might not know all possible factors and physiological conditions that may influence the CNR1 polymorphism. In addition, we revealed a slightly higher incidence of GA genotype and lower incidence of GG genotype in the polymorphism rs806381s in the whole hemodialysis group versus the control group. This result seems to be difficult to be clearly explained based on current knowledge. However, we would like to underline that no difference in the genotype distribution was observed when comparing patients with and without pruritus, which was the main focus of the study.

Based on our study, we suggest that there is no relationship in the CNR1 gene polymorphism and the presence of UP. Due to some limitations of our research such as limited number of patients and the eventual influence of not studied systemic factors, further studies are needed to confirm our results.

## Figures and Tables

**Table 1:** Sequences of CNR1 primers.

Polymorphism	Forward primer (3′-5′)	Reverse primer (3′-5′)
rs12720071	GATGAAGGCTCAGGGTGCTAGAGG	TAGTGCTGTCAGCCCCATTGTCCC
rs806368	GAGACCACCCATATCATGCACACA	AACTCTGATCCCCAGTAGGCCTAG
rs1049353	CCTGCGACACGCTTTCCGGA	CTGCCAGGGAGGCATCAGGC
rs806381	CATGAGCCATGAGGTTTTCT	CATTTGAAGGCCTGTAACTT
rs10485170	TTAACCAATGGTTCATCGTC	ATGTGGTTCTCAGGCATCAG
rs6454674	ATGGAGCCTGTCCTTTAGGT	TATCCAGGAATGCTGCAAAA
rs2023239	CATGAGCCATGAGGTTTTCT	CATTTGAAGGCCTGTAACTT

**Table 2:** Correlation between CNR1 genotype frequencies in patients with and without uremic pruritus.

Polymorphism	Patients without uremic pruritus			Patient with uremic pruritus			*p*
rs12720071	AA	AG	GG	AA	AG	GG	0.64
	65 (78.3%)	15 (18.1%)	3 (3.6%)	62 (81.6%)	13 (17.1%)	1 (1.3%)	

rs806368	TT	CT	CC	TT	CT	CC	0.85
	52 (62.7%)	26 (31.3%)	5 (6.0%)	45 (59.2%)	27 (35.5%)	4 (5.3%)	

rs1049353	AA	AG	GG	AA	AG	GG	0.63
	2 (2.4%)	35 (42.2%)	46 (55.4%)	4 (5.3%)	30 (39.5%)	42 (55.3%)	

rs806381	AA	AG	GG	AA	AG	GG	0.93
	33 (39.8%)	38 (45.8%)	12 (14.5%)	29 (38.2%)	37 (48.7%)	10 (13.2%)	

rs10485170	AA	AG	GG	AA	AG	GG	0.2
	76 (91.6%)	7 (8.4%)	0 (0%)	63 (82.9%)	12 (15.8%)	1 (1.3%)	

rs6454674	TT	GT	GG	TT	GT	GG	0.22
	34 (41.0%)	36 (43.4%)	13 (15.6%)	29 (38.2%)	41 (53.9%)	6 (7.9%)	

rs2023239	TT	CT	CC	TT	CT	CC	0.31
	70 (84.3%)	11 (13.3%)	2 (2.4%)	57 (75.0%)	17 (22.4%)	2 (2.6%)	

**Table 3:** Differences between CNR1 genotype frequencies in all hemodialysis (HD) patients and the control group.

Polymorphism	HD patients			Control group			*p*
rs12720071	AA	AG	GG	AA	AG	GG	0.09
	127 (79.9%)	28 (17.6%)	4 (2.5%)	104 (69.3%)	38 (25.3%)	8 (5.3%)	

rs806368	TT	CT	CC	TT	CT	CC	0.75
	97 (61.0%)	53 (33.3%)	9 (5.7%)	91 (60.7%)	51 (35%)	7 (4.7%)	

rs1049353	AA	AG	GG	AA	AG	GG	0.12
	6 (3.8%)	65 (40.9%)	88 (55.3%)	4 (2.7%)	46 (30.7%)	100 (66.7%)	

rs806381	AA	AG	GG	AA	AG	GG	**0.04**
	62 (39.0%)	75 (47.2%)	22 (13.8%)	53 (35.3%)	59 (39.3%)	38 (25.3%)	

rs10485170	AA	AG	GG	AA	AG	GG	0.26
	139 (87.4%)	19 (12.0%)	1 (0.6%)	124 (82.7%)	26 (17.3%)	0 (0%)	

rs6454674	TT	GT	GG	TT	GT	GG	0.22
	63 (39.0%)	77 (48.4%)	19 (12.0%)	54 (36.0%)	66 (44.0%)	29 (19.3%)	

rs2023239	TT	CT	CC	TT	CT	CC	0.09
	127 (79.9%)	28 (17.6%)	4 (2.5%)	103 (68.7%)	43 (28.7%)	3 (2.0%)	
